# Exosomal crosstalk: the metastatic language of hepatocellular carcinoma

**DOI:** 10.3389/fimmu.2025.1701305

**Published:** 2026-01-06

**Authors:** Qian Xu, Jinhan Chen, Yi Liu, Jinsheng Yu, Fangmin Zhao, Qijin Shu

**Affiliations:** 1 The First School of Clinical Medical, Zhejiang Chinese Medical University, Hangzhou, China; 2 Department of Oncology, The First Affiliated Hospital of Zhejiang Chinese Medical University (Zhejiang Provincial Hospital of Chinese Medicine), Hangzhou, China

**Keywords:** biomarkers, exosomes, hepatocellular carcinoma, intercellular communication, therapeutic targets

## Abstract

Exosomes have garnered considerable attention in hepatocellular carcinoma (HCC) research owing to their critical function in regulating intercellular communication within the tumor microenvironment. Their inherent composition, structural integrity, and cargo-specific properties underpin critical functions in orchestrating cellular information transfer. This review delineates the fundamental biology of exosomes, their multifaceted pathophysiological roles in HCC, and the inherent translational promise of exosome-based therapeutic platforms for HCC management. Emerging evidence positions exosomes as dual-functional entities in HCC: not only propagating tumor progression and metastasis but also emerging as liquid biopsy-based diagnostic indicators for early detection and prognostic stratification, as well as nanoscale delivery platforms for site-specific therapeutic payloads. Nevertheless, despite growing recognition of exosomal functions in HCC, the precise mechanisms governing their functional duality and clinical translatability demand further elucidation. Through critical appraisal of extant literature, this review delineates actionable research priorities to catalyze mechanistic dissection and accelerate bench-to-bedside translation in exosome-based HCC management.

## 1 Introduction

Based on the latest cancer statistics, liver cancer accounts for the sixth position in terms of malignant tumors incidence and ranks third in mortality worldwide. In 2022, there were 865,000 new cases of liver cancer and 758,000 deaths globally, accounting for 4.3% and 7.8% of all malignant tumor incidences and deaths, respectively (1). In China, the incidence rate (12.7/100,000) and mortality rate (10.9/100,000) of liver cancer in males are approximately 2- to 3- fold exceeding those in females, whose incidence rate and mortality rate are 4.8/100,000 and 4.1/100,000, respectively. Primary liver cancers mainly include hepatocellular carcinoma (HCC) (approximately 75% to 85%) and cholangiocarcinoma (approximately 10% to 15%). Chronic hepatitis B virus (HBV) and hepatitis C virus (HCV) infections represent major established etiological drivers of HCC, while other significant risk factors include dietary aflatoxin exposure, obesity, heavy alcohol consumption, tobacco smoking, and type 2 diabetes mellitus (T2DM) (2, 3). High rates of metastasis and recurrence, along with short survival, remain major challenges in clinical practice (4). Studies of tumorigenic mechanisms in HCC have revealed that exosomes, as key mediators, play crucial roles in intercellular signaling, tumor microenvironment formation, and immune evasion processes. Exosomes represent a subclass of nanoscale extracellular vesicles (30–150 nm in diameter) that function as intercellular signaling mediators through the targeted delivery of bioactive cargo (including proteins, nucleic acids, and lipids) to recipient cells, thereby orchestrating critical physiological and pathological processes (5). On this basis, exosomes derived from HCC can regulate the tumor microenvironment, which could be used as promising diagnostic biomarkers and tractable therapeutic targets (6). Consequently, this review summarizes current evidence bridging biological characteristics of exosomes to clinical application in HCC, with the ultimate goal of informing novel diagnostic frameworks and molecularly targeted interventions.

## 2 Overview of exosomes

**Figure 1:**
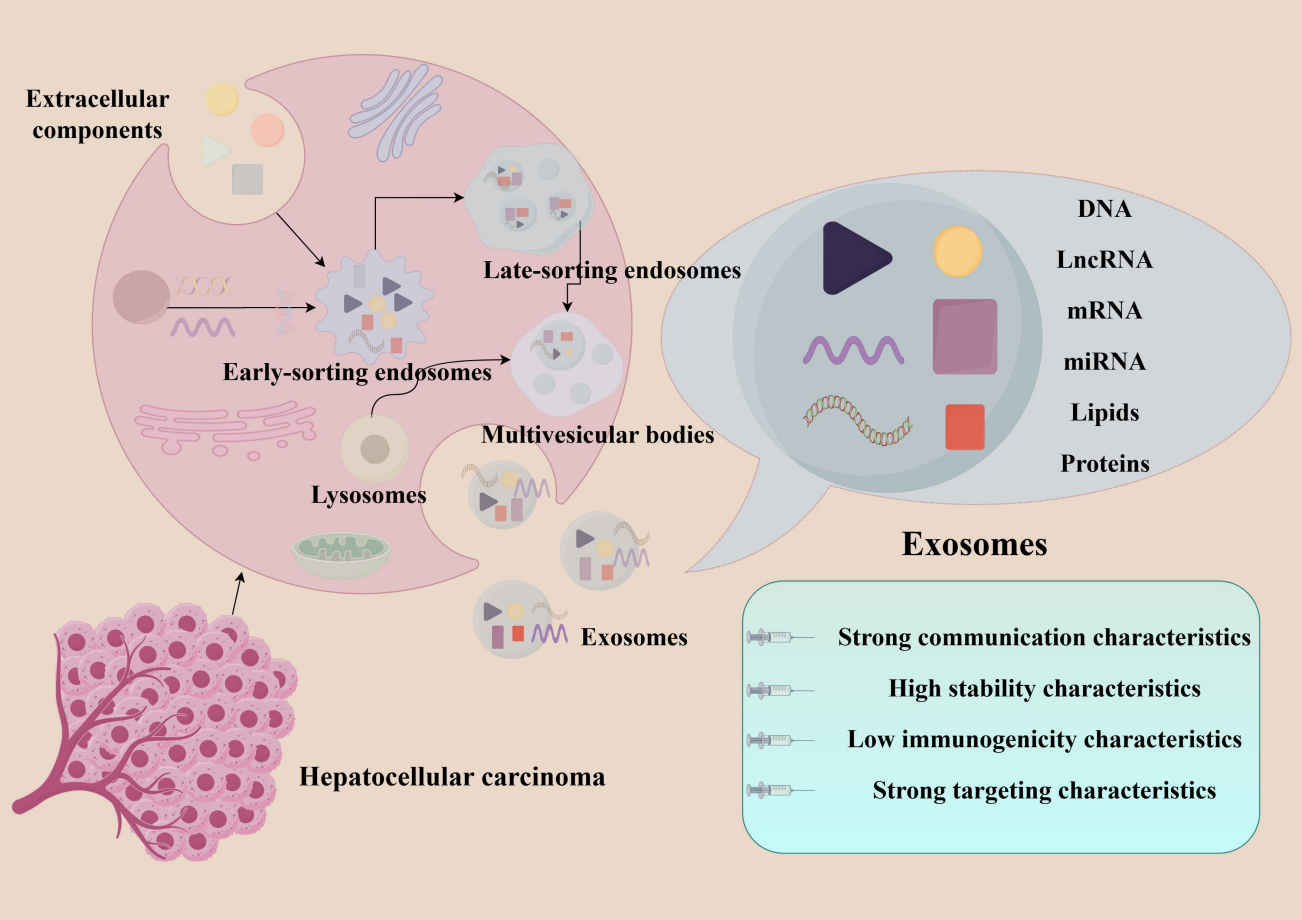
Biogenesis and basic characteristics of exosomes.

Exosomes are extracellular vesicles that possess a lipid bilayer, 30–150 nm diameter, whose seminal discovery emerged from Johnstone’s 1987 pivotal investigation of reticulocyte maturation dynamics (7). Exosome biogenesis constitutes a tightly regulated multistep process. First of all, the cell membrane invaginates, forming a cup-like structure that encloses cell proteins and extracellular components, resulting in the formation of early-sorting endosomes (ESEs). Subsequently, the ESE membrane is concave inward, encapsulating proteins, nucleic acids and metabolites in the cytosol into the ESEs to form late-sorting endosomes (LSEs). Then LSEs mature into multivesicular bodies (MVBs), some of which are degraded by fusion with autophagosomes or lysosomes, while others can fuse with the plasma membrane and release intraluminal vesicles to the extracellular, which are ultimately exosomes (8, 9) (**Figure 1**).

Exosomes are ubiquitous in physiological fluids (e.g., blood, urine, saliva), which contain nucleic acids, proteins, and lipids. Their release could reshape the tumor immune microenvironment, which is regulated by various intracellular and extracellular factors (10–12). Endowed with unique properties, exosomes hold potential as biomarkers and drug delivery vehicles (13–16): (1) intercellular communication: exosomes serve as communication carriers for biological signaling, coordinating cellular processes such as proliferation, differentiation, and senescence; (2) high stability: the phospholipid bilayer structure of exosomes protects their contents from external environment, enabling them to remain stable in body fluids and even traverse certain biological barriers; (3) low immunogenicity: exosomes exhibit excellent biocompatibility and high safety profile; (4) targeted delivery: the components within exosomes can bind to specific surface receptors on target cells, enabling precise targeting to lesion sites.

## 3 Exosomes: active orchestrators in HCC

### 3.1 Exosomes are involved in HCC angiogenesis

HCC is characterized by abundant blood supply, and exosomes are involved in all aspects of angiogenesis in HCC. Tumor-derived exosomes orchestrate pro-angiogenic crosstalk by transferring oncogenic cargoes to vascular endothelial cells, thereby driving neovascularization within the tumor microenvironment (17). HCC-derived exosomes delivered angiopoietin-2 (ANGPT2), which was transported to human umbilical vein endothelial cells (HUVECs) by endocytosis to drive robust pro-angiogenic responses. However, by CRISPR-Cas system knockout, the angiogenesis driven by exosome-borne ANGPT2 could be significantly inhibited, and the activation of epithelial-mesenchymal transition (EMT) in HCC can also be suppressed (18). Rab27A/B orchestrate exosome secretion via governing MVB-plasma membrane docking and fusion, thereby accelerating tumor progression by enhanced delivery of pro-metastatic cargoes. Zhang Z et al. discovered that PRR34-AS1 promoted exosomal secretion of vascular endothelial growth factor (VEGF) and transforming growth factor β (TGF-β) in HCC cells by increasing Rab27A expression. This process enhanced the malignant phenotype of human liver epithelial cells, which propelled HCC proliferation and neovascularization (19). Additionally, hypoxic environment promoted the secretion of exosome glypican-3 (GPC3) by activating hypoxia-inducible factor 1-alpha (HIF-1α). This enhanced HCC oncogenesis through proliferative signaling by Wnt/β-catenin-mediated induction, migratory capacity, and EMT processes. Meanwhile, the angiogenic potential of HUVECs was enhanced, suggesting that hypoxia-induced exosomal factors contributed to angiogenesis of HCC (20). While Yusuke Matsuura (21) detected that miR-155 was also substantially up-regulated in hypoxic conditions, inducing the proliferation of HUVECs. Although the mechanisms have not been further clarified, clinical data from a small sample size showed that in the plasma of HCC patients, the high expression of exosomal miR-155 was significantly associated to recurrence and metastasis after radical surgery. Li Zhang (22) also discovered that miR-155 could promote growth of HCC via the AT-rich interactive domain 2 (ARID2) -mediated AKT phosphorylation pathway. This discovery established a clinically actionable reference for prognostic stratification and survival prediction in HCC patients. XY Huang (23) observed that circRNA-100,338 was markedly upregulated in metastatic HCC, which enhanced the invasion ability of HCC. *In vitro* and *in vivo* analyses demonstrated that exosomal circRNA-100,338 orchestrated pro-angiogenic phenotypes in HUVECs, including enhanced proliferation, vasculogenic mimicry formation, microvascular permeability, and neovascularization. XJ Lin et al. (24) suggested that high level of miR-210 in HCC serum was associated with higher microvascular density in tissues. Exosomal miR-210 can be conveyed to endothelial cells, leading to facilitating tumor angiogenesis via targeting inhibition of sma and mad homolog 4 (SMAD4) and signal transducer and activator of transcription 6 (STAT6). Kai Zhu (25) discovered that miR-146a could mediate breast cancer susceptibility gene 1 (BRCA1) to increase the level of platelet-derived growth factor receptor α (PDGFRA), which was associated with microvascular invasion. However, it is interesting that exosomal miR-451a, acting as a tumor suppressor, was observed to trigger apoptosis in both HCC cell and HUVECs. Furthermore, miR-451a inhibited the migration of HUVECs and changed the permeability of blood vessels, which could potentially reduce tumor angiogenesis (26). SS Dong (27) also found that miR-3682-3p released by HCC cells reduced angiogenesis by targeting angiopoietin-1 to inhibit RAS-MEK1/2-ERK1/2 signaling pathway. In summary, exosomes exert multifaceted core regulatory roles in the angiogenesis process of HCC. Acting as key information carriers, they transport various functional molecules (such as ANGPT2, miR-155, circRNA-100,338, miR-210, etc.) to vascular endothelial cells, thereby promoting endothelial cell proliferation, migration, and neovascularization. Meanwhile, studies have revealed that certain exosomal cargos (e.g., miR-451a, miR-3682-3p) inhibit angiogenesis by triggering endothelial cell apoptosis and targeting angiopoietin-1 to suppress RAS-MEK1/2-ERK1/2 signaling pathways, demonstrating their dual functional nature. These findings not only reveal key mechanisms of exosomes in HCC progression but also provide robust theoretical support for their potential as anti-angiogenic therapeutic targets and prognostic biomarkers.

### 3.2 Exosomes are involved in HCC invasion and metastasis

Exosomes are crucial mediators in the tumor microenvironment, which are involved in intercellular communication, and playing an indispensable role in the invasion and metastasis of HCC. The exosome M2 macrophage polarization associated lncRNA (LncMMPA) was secreted by tumor-associated macrophages (TAMs). It can not only polarize M2 macrophages but also function as a competitive microRNA sponge for miR-548s, leading to the transcriptional upregulation of aldehyde dehydrogenase 1 family member A3 (ALDH1A3). This elevation in ALDH1A3 expression enhanced glucose metabolism and accelerated cellular proliferation in HCC (28). The investigators (29) observed that the expression profile in HCC was characterized by a significant increase of miR-27a-3p and a concomitant decrease in thioredoxin-interacting protein (TXNIP). Furthermore, M2 macrophage-derived exosome miR-27a-3p further affected HCC proliferation, migration, invasion, and drug resistance via targeting TXNIP. XH Zhou (30) found that HCC-derived exosomes induced the overexpression of microRNA-761 (miR-761) in HCC, and reprogrammed the tumor microenvironment via targeting the SOCS2/JAK2/STAT3 pathway, which activated the tumor-associated fibroblasts (CAFs). Consistent with prior evidence (31), lysyl oxidase-like 4 (LOXL4) was significantly upregulated in HCC and correlated with poor clinical prognosis. Mechanistically, exosome-derived LOXL4 orchestrated FAK/Src pathway activation by hydrogen peroxide-mediated signaling, thereby enhancing adhesion, migratory, and invasive potential of tumor cells. Furthermore, HCC-derived exosomes transferred LOXL4 to HUVECs by a paracrine mechanism to enhance tumor angiogenesis. Moreover, exosome-derived miR-375 could promote the proliferation and migration of HCC by targeting insulin growth factor binding protein 4 (IGFBP4) (32). The clinical trial (33) discovered that exosomes derived from S100A4, a metastasis-associated protein, activated the EMT process, consequently accelerating invasive protrusion and metastatic dissemination in HCC. Notably, we uncovered a context-dependent functional duality of exosomes in HCC, where specific subpopulations exerted paradoxical pro-tumorigenic and tumor-suppressive effects through distinct cargo-mediated signaling pathways. Lei Zhang et al. (34) found that macrophages that overexpressing recombination signal binding protein for immunoglobulin kappa J region (RBPJ) secreted exosomal circRNAs, which inhibited the proliferation and metastasis of HCC and promoted apoptosis via the hsa_circ_0004658/miR-499b-5p/JAM3 pathway. YS Ma (35) showed that miR-15a, exosomes derived from mesenchymal stem cells (MSCs), had the capacity to interact with spalt-like transcription factor 4 (SALL4), which could slow the proliferation, migration, and invasive potential of HCC. In normal hepatic tissue, miR-199a-3p ranks as the third most abundant miRNA, yet its expression is markedly downregulated in HCC. Functionally, miR-199a-3p acted as a tumor suppressor by directly targeting multiple oncogenic drivers-including HIF-1α, p21-activated kinase 4 (PAK4), yes-associated protein 1 (YAP1), VEGF-A, hepatocyte growth factor (HGF), and matrix metalloproteinase-2 (MMP2), thereby potently inhibiting HCC proliferation, migration, invasion, and angiogenesis (36–38). In conclusion, exosomes function as pivotal mediators within the tumor microenvironment, playing a central role in HCC invasion and metastasis by transmitting various bioactive molecules. These exosomes originate not only from tumor cells but also from components in the microenvironment, such as TAMs and macrophages. They collectively promote malignant progression of HCC by regulating glucose metabolism, activating some signaling pathways (e.g., FAK/Src, JAM3), influencing EMT, and modulating angiogenesis. Notably, exosomal functions exhibit context-dependent properties: certain exosomal subpopulations (e.g., those carrying miR-15a or miR-199a-3p) demonstrate tumor-suppressive activity by inhibiting proliferation and migration while inducing apoptosis. This dual role highlights the complexity of exosome-mediated signaling in HCC, where functional outcomes may be determined by cargo composition and cellular origin.

### 3.3 Exosomes are involved in HCC immune evasion

Tumor can orchestrate macrophage polarization towards either the antitumor M1 or pro-tumorigenic M2 phenotypes. HCC-derived exosomes orchestrate macrophage activation and polarization towards the pro-tumorigenic M2 phenotype via diverse mechanisms, including transfer of specific cytokines and modulation of signaling pathways. This creates an immunosuppressive microenvironment that facilitates immune evasion (39). LncRNA TUC339, highly abundant in HCC-derived extracellular vesicles, functions as a key regulator in facilitating tumorigenesis and metastatic dissemination. Moreover, it is critically involved in dictating M1/M2 macrophage polarization that may facilitate immune evasion (40). CL Yin (41) reported that HCC-derived exosomes drove polarization towards M2 tumor-associated macrophages (TAMs) by triggering NF-κB signaling pathway and stimulating pro-inflammatory factors secretion. Tumor cell-derived exosomes also serve as vehicles for the delivery of miRNA to macrophages, orchestrating functional reprogramming and phenotypic modulation of recipient cells. For example, exosome miR-23a-3p derived from HCC can upregulate programmed death ligand 1 (PD-L1) expression in macrophages and suppress the functional activity of T cells, which may promote immune evasion (42). Neutrophils are crucial components of the innate immune system, and the study showed that TGF-β and Axl-induced CXCL5 boosted neutrophil infiltration in HCC, which may accelerate tumor growth (43). Natural killer (NK) cells are the first-line effectors in cancer immunosurveillance. The prognostic of patients with HCC was closely related to the quantity of NK cells in both peripheral blood and the tumor microenvironment (44). YL Yang (45) reported that both HBV proteins and nucleic acids are detected in serum exosomes from chronic hepatitis B (CHB) patients, and these exosomes actively transmitted HBV to hepatocytes. And HBV-positive exosomes could hamper the proliferation and survival of NK cells, including cytolytic activity, the production of interferon (IFN)-γ and the responsiveness of the cells to poly (I:C) stimulation. However, LH Lv et al. (46) found out that exosomes from HepG2, which were treated with anticancer drugs, showed increased immunogenicity in triggering NK cells responses specific to heat shock proteins (HSPs), resulting in strong antitumor effects. Consequently, deeper mechanistic investigations between HCC-derived exosomes and NK cell function are necessary, with findings holding significant potential for immunotherapeutic advancement. The activation of regulatory T cells (Tregs) is regarded as one of the most crucial immune escape mechanisms in tumor (47). CircGSE1 drove HCC progression by miR-324-5p/TGFBR1/Smad3 pathway, which upregulated Tregs, and inhibited the activation of CD4^+^ and CD8^+^ T cells (48). XC Wang (49) revealed that 14-3-3ζ, which may be presented to T cells by exosomes, increased in the HCC and suppressed the anti-tumor immunity of tumor-infiltrating lymphocytes (TILs). In addition, NKG2D ligands expressed by exosomes had a strong ability to attenuate the cytotoxic function of NK cells and cytotoxic T cells (50). LS Ye et al. (51) revealed that patients with HCC exhibited a significant enrichment of TIM-1^+^ regulatory B cells (Bregs) within the tumor microenvironment. These infiltrating Bregs were characterized by their robust expression of the immunosuppressive cytokine IL-10 and induced potent suppression of CD8^+^ T cell effector function. Moreover, by the mitogen-activated protein kinase (MAPK) and Toll-like receptor (TLR) 2/4 signaling pathways, exosome-derived high mobility group box 1 (HMGB1) triggered B cells and encouraged TIM-1^+^ Breg cell proliferation. Notably, Quan Rao (52) demonstrated that HCC-derived exosomes paradoxically potentiated dendritic cells (DC) activation and triggered antigen-specific CD8^+^ T cell expansion, suggesting their dual role in modulating antitumor immunity. In summary, exosomes exert multifaceted roles in reshaping the tumor immune microenvironment (**Table 1**). They actively promote macrophages polarization toward the pro-tumor M2 phenotype, attenuate the cytotoxic function of NK cells, enhance the expansion and activation of immunosuppressive cells such as Treg cells and Breg cells, and suppress effector T cell responses. These synergistic effects collectively establish a potent immunosuppressive environment that facilitates tumor immune evasion. Interestingly, we also observed that specific exosome subpopulations, particularly under distinct conditions such as drug treatment or antigen presentation, may paradoxically enhance immune activation, highlighting the complexity of their immunoregulatory functions. A deeper understanding of the mechanisms underlying these processes is crucial for developing novel exosome-based immunotherapy strategies for HCC.

**Table 1:** Regulation of exosomes in the tumor microenvironment.

Exosomal cargoes	Sources	Functions	References
Unclear	HCC	angiogenesis↑	(17, 18)
PRR34-AS1	HCC	proliferation and angiogenesis↑	(19)
GPC3	Unclear	proliferation, migration, EMT, and angiogenesis↑	(20)
miR-155	HCC	proliferation and angiogenesis↑	(21, 22)
circRNA-100,338	HCC	proliferation, migration, and angiogenesis↑	(23)
miR-210	Unclear	angiogenesis↑	(24)
miR-146a	Unclear	angiogenesis↑	(25)
miR-451a	Unclear	angiogenesis↓	(26)
miR-3682-3p	HCC	angiogenesis and EMT↓	(27)
LncMMPA	TAMs	glucose metabolism and cell proliferation↑	(28)
miR-27a-3p	TAMs	cell proliferation, migration, invasion, and drug resistance↑	(29)
miR-761	HCC	invasion and metastasis↑	(30)
Unclear	HCC	cell matrix adhesion, migration, invasion, and angiogenesis↑	(31)
miR-375	Unclear	cell proliferation and migration↑	(32)
Unclear	S100A4	EMT, invasion and metastasis↑	(33)
circRNAs	Macrophages	proliferation and metastasis↓; apoptosis↑	(34)
miR-15a	MSCs	invasion and metastasis↓	(35)
miR-199a-3p	Unclear	migration, invasion and angiogenesis↓	(36–38)
lncRNA TUC339	HCC	cell proliferation and migration↑	(40)
miR-23a-3p	HCC	immune evasion↑	(42)
HBV-positive exosomes	Unclear	NK cells↓	(45)
Unclear	HCC treated with anticancer drugs	NK cells↑	(46)
circGSE1	HCC	Tregs↑; CD4^+^ and CD8^+^ T cells↓	(48)
Unclear	Unclear	TILs↓	(49)
HMGB1	Unclear	TIM-1^+^ Breg cell↑	(51)
Unclear	HCC	DC and CD8^+^ T cell↑	(52)

↑ increase, ↓ decrease.

### 3.4 Exosomes are involved in HCC therapeutic resistance

Sorafenib and Lenvatinib constitute the cornerstone first-line systemic therapies for advanced HCC (53). However, the emergence of drug resistance substantially curtails their therapeutic efficacy and long-term clinical utility. Consequently, emerging researches are focusing on harnessing exosome-based strategies to overcome or delay the acquired resistance in HCC (54). MiR-200c-3p in M2 macrophage exosomes can influence the PI3K/AKT signaling pathway via obstructing the interaction between BAP1 gene and PTEN gene in hepatocytes, which could strengthen the resistance to sorafenib (55). GH Wang et al. (56) discovered that miR-744 was downregulated in exosomes released by HCC tissues and cell lines, which consequently facilitated the proliferation of HCC while reducing the chemosensitivity to sorafenib. Sorafenib significantly modulated the miRNA expression profile in HCC, altering the abundance of at least 11 specific miRNAs (57). This modulation had dualistic functional consequences: tumor-suppressive miRNAs, like miR-200c-3p and miR-27a-3p, were upregulated, inhibiting migration and invasion. Meanwhile, sorafenib increased the expression of oncogenic miRNAs, including miR-122-5p, miR-148b-3p, miR-194-5p, miR-222-5p, and miR-512-3p, which collectively enhanced tumorigenicity by driving proliferative signaling, promoting invasiveness, and conferring resistance to apoptosis. MB Cao (58, 59) discovered that exosome-derived lnc-FAM72D-3 changed the cytoskeleton of HCC by the MBNL1/FAK pathway and hsa_circ_0007132 inhibited the ubiquitin-mediated degradation of NONO, which both increased resistance of Lenvatinib. XH Yao et al. (60) showed that miR-9 facilitated EMT and bolstered the migratory and invasion HCC cells. Concurrently, miR-9 had the potential to promote the resistance to anti-angiogenesis therapy via the secretion of VEGF-exosomes mediated by tumor-associated endothelial cells (TAECs). XC Wei et al. (61) indicated that hepatitis B core antigen (HBc) upregulated the expression of miR-135a-5p, which was highly enriched in HCC tissues, and directly targeted vesicle-associated membrane protein 2 (VAMP2). Through the newly identified miR-135a-5p/VAMP2 signaling pathway, HBc promoted proliferation and induced chemoresistance in HCC. Taken together, sorafenib and Lenvatinib are first-line treatments for advanced HCC, but acquired resistance severely limits their clinical efficacy, which may be potentially involved in exosome-mediated mechanisms. Exosomes transport specific miRNAs (e.g., miR-200c-3p, miR-744) and lncRNAs (e.g., lnc-FAM72D-3, hsa_circ_0007132) that activate pro-tumor signaling pathways, and inhibit drug-induced apoptosis. Therefore, targeting their biogenesis or receptor-mediated delivery pathways offers a promising avenue to overcome current therapeutic bottlenecks and propel the clinical translation of precision-guided resistance reversal strategies.

### 3.5 Exosomes are used as biomarkers for diagnosis and prognosis

**Figure 2:**
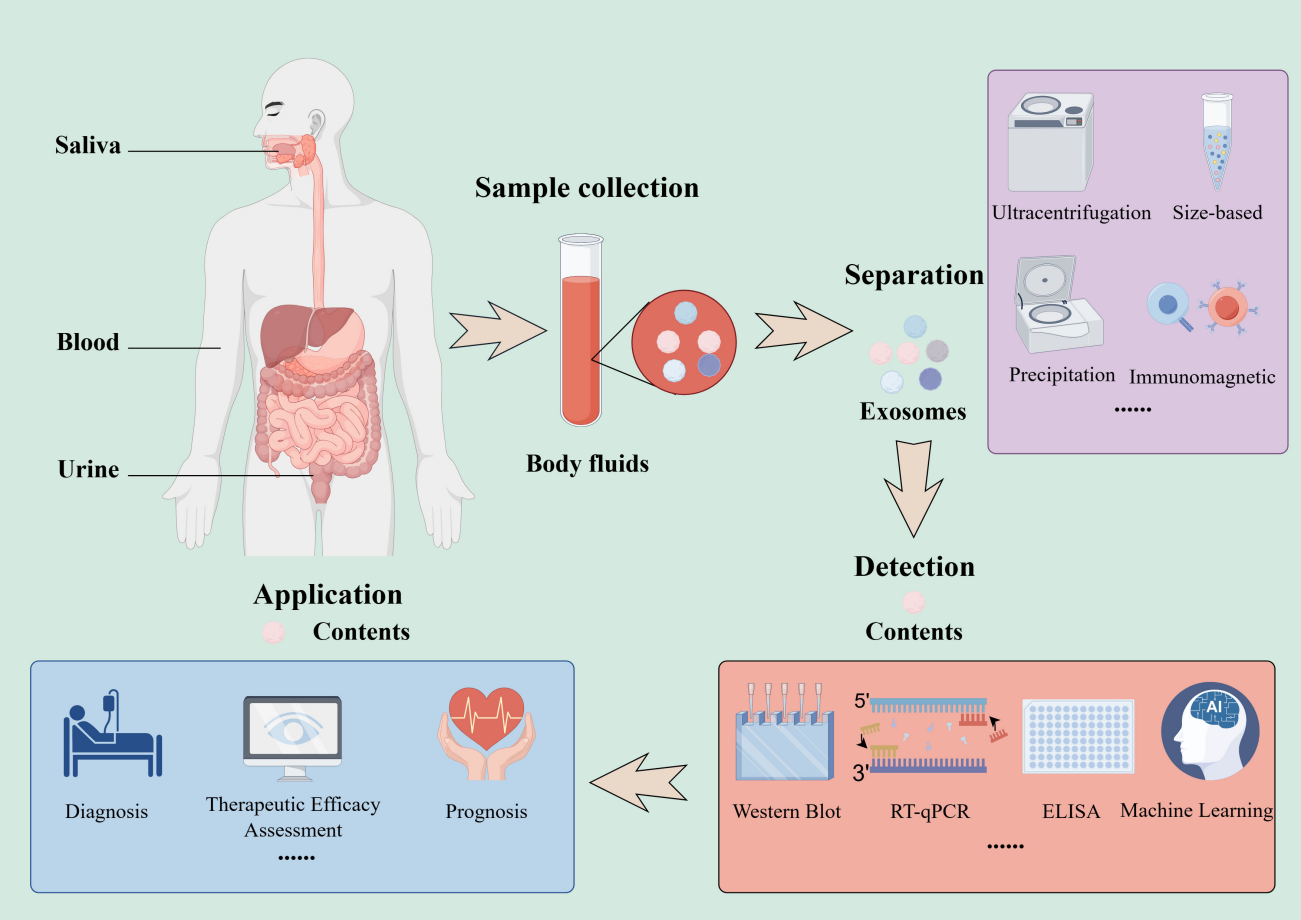
Exosomes as biomarkers: from liquid biopsy to clinical application.

Current researches are intensively investigating exosome-derived molecular biomarkers that exhibit HCC-specificity and dynamically reflect disease progression, metastasis, and prognostic (**Table 2**). Based on liquid biopsy technology, these exosomes hold significant potential in early diagnosis, therapeutic efficacy monitoring, and prognosis assessment (75, 76) (**Figure 2**). XQ Ding (77) deciphered the diagnostic-prognostic value of exosome-associated gene signatures in nonalcoholic fatty liver disease (NAFLD)-driven HCC, offering new biomarkers for prognosis management of NAFLD-HCC patients. JC Wang (62) indicated that the exosomal lncRNA SENP3-EIF4A1 in the plasma of HCC patients was markedly lower compared with the healthy individuals. And SENP3-EIF4A1 could be transferred to HCC cells via exosomes, which may stimulate apoptosis while reducing HCC invasion and metastasis. So SENP3-EIF4A1 hold potential as a promising novel biomarker for the clinical detection of HCC. Peng Deng (63) showed that serum exosomal miR-122 and miR-148a were lower in HCC. This downregulation was negatively correlated with clinical stage and lymph node metastasis, while it showed a positive association with the level of tumor differentiation, patient survival rates, and the concentrations of serum markers associated with HCC. Suehiro T et al. (64) suggested that levels of exosomal miR-122 may function as indicators of hepatic injury and remaining liver functionality. Their findings revealed a marked downregulation of miR-122 by post-transarterial chemoembolization (TACE), particularly among patient with liver cancer. And a lower miR-122 ratio has been related to a poor prognosis. Patricia de la Cruz-Ojeda (57) demonstrated that the level of circulating miR-200c-3p was relevant to improved survival, while high concentrations of miR-222-5p and miR-512-3p were correlated with unfavorable prognostic indicators. XF Xue (65) also revealed that the serum of HCC patients had different levels of miR-122, miR-125b, miR-145, miR-192, miR-194, miR-29a, miR-17-5p, and miR-106a compared with normal controls, which could act as diagnostic biomarkers of HCC. MiR-106a was a prognostic factor for HCC that regulated MAPK and JNK pathways to promote tumorigenesis. Dan Rao (66) and HW Wu (67) both reported that the poor prognosis was closely related to the serious upregulation of miR-425-5p in HCC tissues. So, we could prevent HCC migration and proliferation by knocking out miR-425-5p. We mentioned above that miR-199a-3p was expressed in normal liver tissue but was declining in HCC, which may also be correlated with poor prognosis (68). In the study of YX Xu team (69), low level of miR-451a and high level of a disintegrin and metalloprotease 10 (ADAM10) may indicate a poor prognosis of HCC patients. MiR-136-5p and MMP2 were two crucial players in HCC metastasis in the rescue experiments. Patients who had low level of miR-136-5p and high levels of circ_MMP2 or MMP2 may have poor overall survival (70). Furthermore, miR-21 (71), miR-26a (72), miR-146a (25), miR-155 (23, 73), miR-182, and miR-331-3p (74) have been shown to link to the diagnosis or prognosis of HCC. Taken together, exosomes and their contents are stable in body fluids, which can reflect the state of the source cells and the tumor microenvironment. Consequently, they may serve as the promising markers for diagnosis, prognosis, and monitoring, as well as novel targets for future treatments.

**Table 2:** Key exosomal non-coding RNAs in HCC: expression and clinical value.

Type	Molecular name	Expression in HCC	Clinical value	Reference
lncRNA	SENP3-EIF4A1	↓	Diagnostic	(62)
miRNA	miR-122	↓	Diagnostic; Prognostic	(63, 64)
miRNA	miR-148a	↓	Prognostic	(63)
miRNA	miR-200c-3p	/	Prognostic	(57)
miRNA	miR-222-5p	↑	Prognostic	(57)
miRNA	miR-512-3p	↑	Prognostic	(57)
miRNA	miR-125b	↑	Diagnostic	(65)
miRNA	miR-145	↑	Diagnostic	(65)
miRNA	miR-192	↑	Diagnostic	(65)
miRNA	miR-194	↑	Diagnostic	(65)
miRNA	miR-29a	↑	Diagnostic	(65)
miRNA	miR-17-5p	↑	Diagnostic	(65)
miRNA	miR-106a	↑	Diagnostic; Prognostic	(65)
miRNA	miR-425-5p	↑	Prognostic	(66, 67)
miRNA	miR-199a-3p	↓	Prognostic	(68)
miRNA	miR-451a	↓	Prognostic	(69)
miRNA	miR-136-5p	↓	Prognostic	(70)
miRNA	miR-21	↑	Prognostic	(71)
miRNA	miR-26a	↓	Prognostic	(72)
miRNA	miR-146a	↑	Prognostic	(25)
miRNA	miR-155	↑	Prognostic	(23, 73)
miRNA	miR-182	↑	Diagnostic; Prognostic	(74)
miRNA	miR-331-3p	↑	Diagnostic; Prognostic	(74)

↑ increase, ↓ decrease.

Synthesizing these lines of evidence, we propose that exosomes may represent a “double-edged sword” in HCC. The function of exosomes in HCC is determined by multiple interrelated factors: cellular origin, tumor microenvironment, and specific cargo loading mechanisms. If the parent cells are malignant tumor cells or TAMs, they could impose fundamental functional biases on the exosomes they generate. Secondly, dynamic conditions within the TME, such as hypoxia, nutrient deprivation, or therapeutic stress, can reprogram exosome biosynthesis and selectively enrich specific pro-tumor signals. Finally, active sorting mechanisms controlled by molecules like nucleic acids or proteins provide the ultimate specificity, ensuring precise loading of pro- or anti-tumor signals. The complex interplay between cellular origin, tumor microenvironment, and molecular sorting mechanisms ultimately determines the dual role of exosomes.

## 4 HCC therapeutic revolution: synergistic paradigms for advanced therapeutics

### 4.1 Personalized treatment

Exosomes can effectively delay clinical resistance to sorafenib. Treatment with miR-744-enriched exosomes markedly suppressed HCC cell proliferation, overcame chemoresistance, and restored sorafenib sensitivity (56). Human cerebral endothelial cell-derived exosomes carrying elevated miR-214 (hCEC-Exo-214) have been demonstrated to reduce the levels of P-glycoprotein (P-gp) and splicing factor 3B subunit 3 (SF3B3) in HCC when combined with oxaliplatin and sorafenib. It could result in synergistic anti-tumor effects (78). Exosomes, as promising radiosensitizers, can synergize with radiotherapy to overcome radio resistance and potentiate tumoricidal efficacy. In HCC, high expression of exosomal circTMEM56 enhanced radiotherapy response via activating cGAS-STING pathway, driving tumor microenvironment reprogramming (79). Radiotherapy could result in the release of exosomes with elevated Maspin expression, which markedly suppressed tube formation and vascular endothelial growth in HUVECs. MiR-151a-3p suppressed p53 signaling pathway, triggered EMT, and promoted tumor growth, which was significantly reduced after radiotherapy (80). There is evidence that exosomes harboring PD-L1 can replicate the effect of cell-surface PD-L1, it may be used as a therapeutic adjuvant by decreasing exosomal PD-L1 to increase the effectiveness of PD-1/PD-L1 therapy in cancer (81). Liang Cheng (82) confirmed that exosomes from melatonin-treated HCC cells reprogrammed macrophages by reversing their immunosuppressive phenotype and promoting pro-inflammatory activation via the STAT3 pathway, which may offer a novel strategy to alleviate immunosuppression.

### 4.2 Targeted delivery vehicles

**Figure 3:**
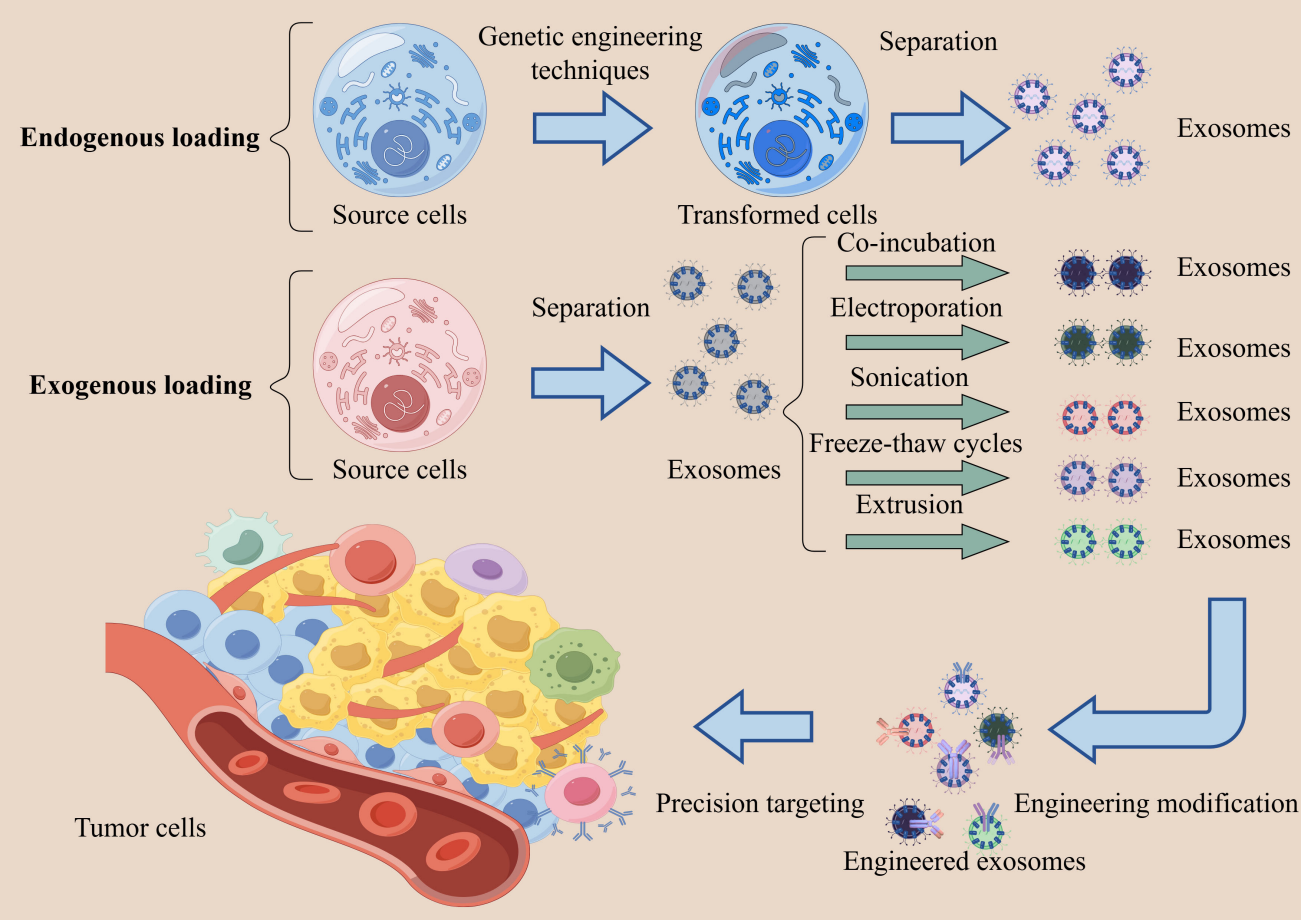
Exosome-mediated precision targeted therapy. There are two primary methods for loading drugs into exosomes. Endogenous loading: cells are transfected using genetic engineering techniques to express the desired proteins, nucleic acids, or drug molecules, followed by the isolation of exosomes containing the bioactive substances. Exogenous loading: First, separate exosomes. Then introduce drugs or bioactive substances using methods such as co-incubation, electroporation, sonication, freeze-thaw cycles, and extrusion. Subsequently, the surface of exosomes is engineered to display targeting ligands (e.g., peptides, antibodies, aptamers, and lipids), enabling precision delivery to specific cells or tissues.

Furthermore, exosomes can function as drug delivery vehicles, enhancing drug targeting and stability (**Figure 3**). Mesenchymal stem cell (MSC)-derived exosomes can be used as clinically actionable natural nanocarriers for targeted molecular delivery. Specifically, exosomes from adipose tissue-derived MSCs could transport miR-199a-3p to HCC cells, which can increase the chemosensitivity to doxorubicin (83). Exosomes that are selectively targeted to HCC (ExoSP94-Lamp2b-RRM) could transport the multiplex small interfering RNA (multi-siRNA) to HCC tissues, which may improve the sorafenib-induced ferroptosis, and thus boost HCC sensitivity to sorafenib (84). Plant-derived exosomes, as emerging anticancer agents, enable targeted therapeutic delivery due to their inherent immunogenicity, biocompatibility, and cell-free nanostructure characteristics. For example, cannabidiol-enriched extracellular vesicles (CBD-EVs) significantly induce mitochondrial apoptosis in HCC cells, evidenced by G0/G1 cell cycle arrest and dose-dependent activation of the Bax/Bcl-2/caspase-9 signaling axis (85).

### 4.3 Exosome vaccines

Exosomes released by HCC cells have soluble immunoregulatory compounds that severely restrict the lymphocyte proliferation. The cytotoxic effect of NK cells was significantly increased by MS-275 edited exosomes, by raising the production of major histocompatibility complex class I polypeptide-related sequence A (MICA), MICB, and HSP 70. It suggested that exosomes created by tumor cells treated with histone deacetylase inhibitor (HDACi) could be used as possible tumor vaccines against HCC (86). When compared with using exosomes or IL-12 alone, the combination of DC-Tumor derived exosomes (TEX) and IL-12 was superior in promoting T lymphocyte proliferation, releasing IFN-γ, and increasing cytotoxicity. Furthermore, the addition of IL-12 made up for the decreased IL-2 secretion by DCs driven by Tex (87). In mice of HCC, DC-derived exosomes (DEX) particularly increased DCs recruitment, accumulation, and activation, which led to enhanced tumor neoantigen cross-presentation and immune response (88, 89). By the use of an exosomal anchor peptide, BF Zuo (90) exhibited that TEXs painted with the functional domain of HMGN1 (TEX-N1ND) enhanced DC immunogenicity, potentially activating T cells and improving vaccine effectiveness. Exosomes, as novel sources of antigens, may offer new ideas for exosome-based cellular immunotherapy.

### 4.4 Engineered exosomes

SN Huang (91) discovered that the GPC3 scFv engineered and IR780-loaded exosomes (IR780@GPC3-EXOs) could quickly target HCC and cause notable tumor suppression by photothermal effects after near infrared (NIR) light. XQ Jia (37) designed a lentiviral vector for pre-miR-199a expression (Lenti-miR-199a), which can delay the proliferation of HCC both *in vitro* and *in vivo* via downregulating HIF-1α. Sushrut Kamerkar (92) designed the engineered exosome delivering an antisense oligonucleotide (ASO) targeting STAT6 (exoASO-STAT6), which selectively targeted tumor macrophages and produced potent antitumor activity. Silvia Baldari (93) noticed the delivery of miR-125b-loaded EVs made in engineered ASCs specifically decreased HCC cell proliferation *in vitro*, which was related to the p53 signaling pathway.

Collectively, exosomes offer transformative strategies to overcome therapeutic bottlenecks in HCC through a synergistic functional triumvirate: precise targeted delivery, immune potentiation, and closed-loop feedback regulation. Currently, the scalable production of exosomes faces significant challenges, including low yields of natural exosomes, high costs of engineered processes, and difficulty in maintaining biological activity. However, emerging strategies such as bioreactors, 3D cell culture models, and genetic engineering techniques offer new avenues (94, 95). Additionally, establishing standardized production protocols and strict quality control standards (e.g., purity and bioactivity) is equally critical, as these are prerequisites for scaling production and eventual clinical approval.

### 4.5 Clinical trials of exosomes in HCC

At present, there are no approved exosome-related products on the market, but researches on the application of exosomes are in full swing. Here, we list the latest clinical trials about exosomes in HCC for further research (**Table 3**).

**Table 3:** Latest clinical trials about exosomes in HCC.

Intervention	Registration number	Country	Conditions	Status	Study type
CDK-004	NCT05375604	United States	Advanced HCC, Gastric Cancer Metastatic to Liver, Colorectal Cancer Metastatic to Liver	Terminated	Interventional
ELUCIDATE	NCT06342414	United States, Japan	HCC, Intrahepatic Cholangiocarcinoma, Cholangiocarcinoma, Primary Liver Cancer, Primary Liver Carcinoma, Hepatic Cancer, Hepatic Carcinoma	Recruiting	Observational
CTC PD-L1, exosomal PD-L1, and exosomal LAG-3 detection	NCT05575622	China	HCC	Unknown	Observational
NA	ChiCTR2400082483	China	HCC	Recruiting	Prognosis study
NA	ChiCTR2200065653	China	HCC	Not yet recruiting	Diagnostic test
NA	ChiCTR-RON-17010895	China	HCC	Recruiting	Cause/Relative factors study

## 5 Conclusion

Currently, HCC remains a global health burden, whose clinical management facing multiple challenges such as high rates of late-stage diagnosis, limited treatment options, and strong treatment resistance. There is an urgent need to explore novel diagnostic and therapeutic strategies to meet clinical demands. Exosomes, as crucial mediators of intercellular communication, play multiple roles in the occurrence, progression and metastasis of HCC. Tumor-derived exosomes exert essential functions in remodeling the tumor microenvironment, affecting tumor angiogenesis, facilitating immune evasion, and conferring therapy resistance. Exosomes represent a “double-edged sword” in HCC: while inherently facilitating immune evasion, they can be engineered as powerful weapons to overcome therapeutic barriers. Exploiting their unique properties, exosomes serve as liquid biopsy diagnostic tools for early detection, dynamic biomarkers for prognostic monitoring, drug delivery vehicles for targeted therapy, and other emerging applications. Future research will focus on: (1) optimizing exosome-based diagnostic and prognostic prediction systems by screening highly specific biomarker combinations from multi-omics data and developing precise prognostic assessment models; (2) advancing precision exosome-mediated targeted therapy through improved drug delivery systems and exploration of combination treatment strategies; (3) promoting the clinical translation and standardization of exosome technology by enhancing preclinical safety and efficacy validations and establishing standardized isolation and production protocols. Collectively, exosomes exhibit great potential in HCC research and therapeutics. By delving deeper into their biological properties and clinical applicability, we can advance early detection, prognostic evaluation, and targeted therapy of HCC, ultimately enabling more precise and effective personalized regimens.
